# Construction and Validation of an Epigenetic Regulator Signature as A Novel Biomarker For Prognosis, Immunotherapy, And Chemotherapy In Hepatocellular Carcinoma

**DOI:** 10.3389/fimmu.2022.952413

**Published:** 2022-07-14

**Authors:** Jialiang Cai, Suiyi Wu, Feng Zhang, Zhi Dai

**Affiliations:** ^1^ Zhongshan Hospital, Liver Cancer Institute, Fudan University, Shanghai, China; ^2^ State Key Laboratory of Genetic Engineering, Fudan University, Shanghai, China; ^3^ Key Laboratory of Carcinogenesis and Cancer Invasion, Ministry of Education, Fudan University, Shanghai, China; ^4^ Department of Gastroenterology and Hepatology, Zhongshan Hospital, Fudan University, Shanghai, China

**Keywords:** hepatocellular carcinoma, immunotherapy, chemotherapy, biomarker, prognosis

## Abstract

**Background:**

Epigenetic modification regulates various aspects of cancer biology, from tumor growth and invasion to immune microenvironment modulation. Whether epigenetic regulators (EGRs) can decide tumor malignant degree and risk of immune evasion in liver hepatocellular carcinoma (LIHC) remains unclear.

**Method:**

An EGR signature called “EGRscore” was constructed based on bulk RNA-seq data of EGR in hepatocellular carcinoma (HCC). The correlation between EGRscore and overall survival (OS) was validated in HCC cohorts and other tumor cohorts. Mutation profiles, copy number alterations (CNAs), enriched pathways, and response to immunotherapy and chemotherapy were compared between EGRscore-high and EGRscore-low patients.

**Results:**

We found that EGRscore was associated with OS in HCC as well as several tumors including glioma, uveal melanoma (UVM), and kidney tumors. A mechanism study demonstrated that the distinct mutation profile of TP53 was present in EGRscore-high and EGRscore-low patients. Meanwhile, EGRscore-low patients were characterized with immune cells that promote killing tumors. Furthermore, EGRscore was associated with genes regulating drug resistance in HCC. Finally, we indicated that EGRscore-low patients had higher response rates to immunotherapy and targeted therapy.

**Conclusions:**

EGRscore could be used to distinguish OS, tumor progression, mutation pattern, and immune microenvironment. The present study contributes to improving hepatocellular carcinoma patient prognosis and predicting response to immunotherapy.

## Introduction

The incidence of HCC ranks sixth and that of the tumor-related death ranks third in the world (1, 2). The etiologies for HCC include chronic infection with hepatitis B virus and hepatitis C virus, metabolic liver diseases, and alcohol addiction, which lead to the accumulation of somatic genetic variation and epigenetic modification, and finally contribute to hepatocarcinogenesis (3). Despite improvements in surgical techniques, radiotherapy, and systemic therapy, HCC patients still presented a 5-year survival rate of only 14.1% in China (4). Immunotherapy plays an increasing role in systemic therapy for HCC; however, it could not achieve a high response rate in the clinic. Therefore, unraveling the genomic properties underlying HCC and identifying prognostic markers for HCC are important for improving current treatment approaches and extending the survival of patients.

Epigenetics was originally proposed to define heritable changes in a cellular phenotype that were independent of alterations in the DNA sequence (5). It generally refers to covalent modifications made to histone proteins and nucleic acids (such as DNA methylation, m6A, histone methylation, m1C, and histone acetylation), which cooperatively regulate chromatin structure and gene expression (6). Moreover, epigenetic alternations regulate various aspects of cancer biology, from tumor growth and invasion to immune microenvironment modulation (7). Epigenetic regulators (EGRs), including the enhancer of zeste 2 polycomb repressive complex 2 subunit EZH2 and the methyltransferase SUV39H2, are reported to mediate the modification function of epigenome. In the last decade, epigenetics-based and EGR-based diagnostic and prognostic tools have made great contributions to precision oncology. Notably, diagnostic screens based on DNA methylation have already been applied in the clinic (8). However, the predictive values of EGR-related genes for prognosis and treatment responses in HCC patients have not been fully elucidated.

Immune checkpoint therapy plays an increasingly important role in HCC. However, only a few patients can benefit from it because of the low response rate. Therefore, it is necessary to find a sensitive biomarker to distinguish whether a patient benefits from the immune checkpoint therapy or not. Several biomarkers emerge as to be candidate such as TMB (9), MSI (10), PDL-1 (11). However, all of them had a disappointing prediction effect in HCC (12). Thus, it is urgent to find a precise and sensitive biomarker.

Therefore, the purpose of this study is to deepen our understanding of the underlying mechanisms and functions of EGR-associated gene changes in HCC. Furthermore, an EGR-related prognostic signature was constructed through systematic analysis, which facilitates the screening of patients for immunotherapies and targeted therapies as well as individual prognosis prediction in HCC.

## Method

### Date Access

Data were collected from three independent databases, namely, The Cancer Genome Atlas (TCGA) database (https://portal.gdc.cancer.gov), the International Cancer Genome Consortium (ICGC) database (https://icgc.org/), and the Gene Expression Omnibus (GEO) database (https://www.ncbi.nlm.nih.gov/geo/) for the following tumors: liver hepatocellular carcinoma (LIHC), uveal melanoma (UVM), brain lower grade glioma (LGG), kidney renal papillary cell carcinoma (KIRP), and kidney renal clear cell carcinoma (KIRC). The institutional ethics committee waived the ethical approval because the data were obtained from the public database, and all patients’ identities were cancelled. Specific information of associated cohorts is listed in **Supplementary Material 1**.

### Identification of EGRscore Signature

The bulk RNA-sequence data of EGRs were collected, including 40 histone acetylation regulators, 34 histone methylation regulators, 5 DNA methylation regulators, 23 m6A methylation regulators, 15 m5C regulators, and 9 m1C regulators. All genes are listed in **Supplementary Material 2**. The “limma” R package was performed to screen RNA-seq data for differentially expressed genes (DEGs) between HCC and normal patients (13). The selection criteria were as follows: false discovery rate (FDR) < 0.05, and |log2 fold change| > 0.67. Then, EGRscore signature was constructed using the least absolute shrinkage and selection operator (LASSO) machine learning algorithm. Finally, 6 EGRs were contained in the EGRscore signature, including EZH2, TRMT6, YBX1, IGF2BP3, SUV3H2, and YTHDF1.

### Receiver Operating Characteristic (ROC) Analysis

The “timeROC” package was applied to calculate the 1-, 2-, and 3-year survival and the “pROC” package was used to analyze the response to immunotherapy based on the bulk RNA-sequence data. Diagnostic accuracy of the EGR signature was evaluated by area under the curve (AUC).

### RNA Isolation and qRT-PCR

Total RNA from cultured cells was extracted using Trizol (Invitrogen) according to the manufacturer’s instructions. cDNAs were generated using Hifair^®^ II 1st Strand cDNA Synthesis SuperMix (Yeasen, China) with random primer and qPCR reactions were carried out using the Maxima SYBR Green qPCR Master Mix (Thermo Scientific). 18S mRNA were examined as an internal control for normalization. Gene expression changes relative to GAPDH were calculated using the ΔΔCT method.

The sequence of primers were as follows: Human 18S: forward, 5’-CGGCGACGACCCATTCGAAC-3’, reverse, 5’-GAATCGAACCCTGATTCCCCGTC-3’; EZH2: forward, 5’-GTACACGGGGATAGAGAATGTGG-3’, reverse, 5’-GGTGGGCGGCTTT5’-TATATCGGAAACCTCAGCGAGA-3’, reverse, 5’-GGACCGAGTGCTCAACTTCT-3’; TRMT6: forward, 5’-GGTGCTGAAACGTGAAGATGT-3’, reverse, 5’-CTTGGGCTGTAGACTTCCTCC-3’; YTHDF1: forward, 5’-ATACCTCACCACCTACGGACA-3’, reverse, 5’-GTGCTGATAGATGTTGTTCCCC-3’; SUV39H2: forward, 5’-TCTATGACAACAAGGGAATCACG-3’, reverse, 5’-GAGACACATTGCCGTATCGAG-3’; YBX1: forward, 5’-GGGGACAAGAAGGTCATCGC-3’, reverse, 5’-CGAAGGTACTTCCTGGGGTTA-3’.

### Functional Enrichment Analysis

Kyoto Encyclopedia of Genes and Genomes (KEGG) analysis was performed to annotate the genes using the “clusterProfiler” R package (14). The thresholds were set as follows: *p*-value < 0.05, *q*-value < 0.05. Hallmark gene sets were downloaded from the MSigDB database (https://www.gsea-msigdb.org/gsea/index.jsp) and manipulated in the GSEA software (v4.2.0) (15).

### Estimation of Tumor immune Infiltration Cell Types and Associated Function

We implemented a single-sample GSEA (ssGSEA) method using gene set variance analysis (GSVA) and the “GSEABase” R package to analyze tumor immune infiltration cell types. Wilcoxon test was applied to analyze immune-infiltrating cells and associated functions between the EGRscore-high patients and EGRscore-low patients.

### Statistical Analysis

Statistics analysis was calculated by R (version 4.1.2, www.r-project.org), Perl language, and GraphPad Prism (https://www.graphpad.com/) using two-tailed unpaired Student’s *t*-test or log-rank test, unless otherwise specified. Elimination of invalid clinical information for patients was achieved through Microsoft Office (https://products.office.com/zh-cn/home). Wilcoxon test was performed when the data were not normally distributed. Chi-square test was performed to compare the difference of response rates for immunotherapy between the EGRscore-high and EGRscore-low group. Fisher’s exact test and Pearson’s Chi-square test are applied respectively when applicable. *p* < 0.05 was considered as statistically significant.

## Results

### Constructions of an Epigenetic Regulator Signature

**Figure 1:**
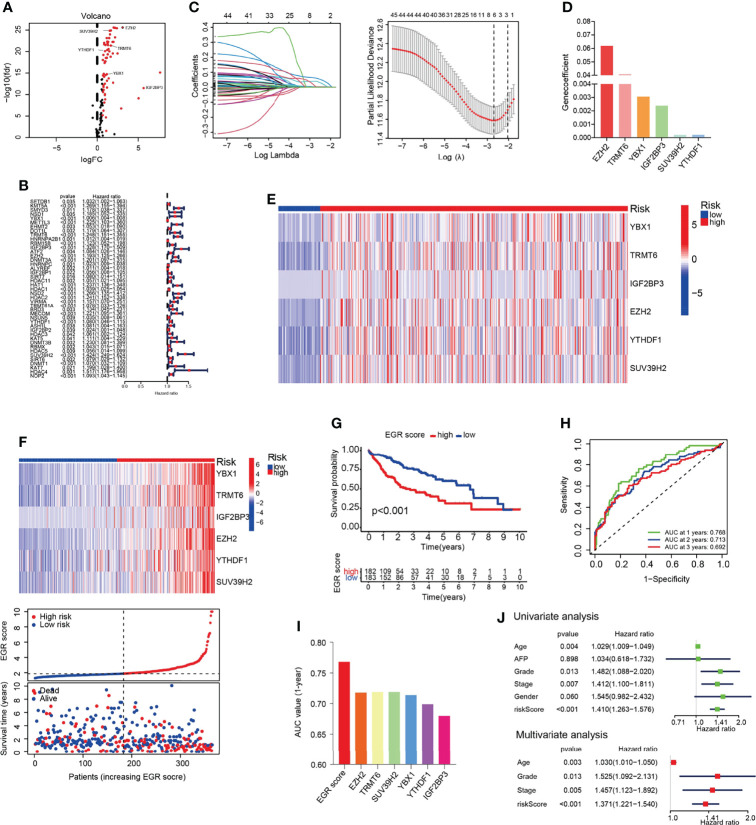
Construction of EGR signature in the TCGA-LIHC cohort. **(A)** Volcano plot of the epigenetic regulators. **(B)** Univariate Cox analysis of the genes selected by DEGs. **(C)** Cross-validation for tuning the parameter selection in the LASSO regression. **(D)** The coefficients of the 6 OS-related genes. **(E)** Heatmap of genes consisted in EGR signature in normal and HCC patients. **(F)** Heatmap of genes consisted in EGR signature, the distribution of patients, and the survival status for each individual in low-risk and high-risk HCC patients. **(G)** Kaplan–Meier curves for the OS of patients between the high- and low-risk groups. **(H)** Time-dependent ROC curves demonstrated the predictive efficiency. **(I)** Area under the ROC curve of 1-year survival. **(J)** Univariate and multivariate Cox regression analyses for the EGRscore.

The data of 374 HCC patients and 50 normal patients were collected from TCGA. EGRs including 116 genes were selected, and the result indicated that 67 EGRs were upregulated significantly in HCC following the standard *p* < 0.05 and |log2FC| > 0.67 (**Figure 1A**). Then, univariate Cox analysis was performed, and 44 differentially expressed EGRs associated with overall survival (OS) (*p* < 0.05) were selected (**Figure 1B**). Subsequently, we used LASSO-penalized Cox regression and six genes were selected to construct a Cox proportional hazards regression signature (**Figure 1C**). The risk score for predicting survival time was calculated with the following formula based on the 6 genes: risk score = (0.061849 × EXPEZH2) + (0.002392 × EXPIGF2BP3) + (0.040777 × EXPTRMT6) + (0.000212 × EXPSUV39H2) + (0.003057 × EXPYBX1) + (0.000217 × EXPYTHDF1) (**Figure 1D**). Each patient had an EGRscore according to the formula and the patients were divided into the EGRscore-high group and EGRscore-low group based on the median value.

The heatmap showed that those six genes had a higher expression in HCC patients than normal patients (**Figure 1E**). The results demonstrated that those six genes had a higher expression in the EGRscore-high than the EGRscore-low group (**Figure 1F**). K-M analysis was performed and the result demonstrated that patients with low EGRscore had better survival rate (**Figure 1G**). Then, we performed ROC curve, and the result demonstrated that the AUC values of 1-, 2-, and 3-year OS were 0.768, 0.713, and 0.692, respectively (**Figure 1H**). Moreover, we drew the ROC curve and compared the AUC of EGRscore to each EGR in our signature. The AUC of 1-year OS for the EGRscore signature was 0.768, which was larger than that of each EGR alone (**Figure 1I**). Furthermore, we compared the predictive ability of EGRscore with clinical indicators and the result indicated that EGRscore had the best performance (**Figure S1A**). To explore whether EGRscore could be an independent prognostic factor, we applied the univariate and multivariable Cox regression models. In the TCGA-LIHC cohort, univariate analysis showed that grade, stage, age, and EGRscore were associated with the prognosis (**Figure 1J**). Moreover, in the multivariable model, the result indicated that the EGRscore could act as an independent predictor for prognosis (HR for EGRscore: 1.371, 95% CI: 1.221–1.54, *p* < 0.001, **Figure 1J**). A nomogram was constructed to accurately predict 1-, 3-, and 5-year OS in HCC patients. As independent prognostic factors calculated *via* multivariate analysis, tumor stage, age, grade, and EGR signature were used to construct the nomogram (**Figure S1B**).

In order to verify the correlation of EGR signature and clinical features, we applied diff analysis and survival analysis. The results showed that Stages III and IV, Grades 3 and 4, T3 and 4 stage, vascular invasion, and high expression of AFP (AFP > 400) had a higher EGRscore compared with Stages I and II, Grades 1 and 2, T1 and 2 stage, no vascular invasion, and low expression of AFP (AFP ≤ 400), respectively (**Figures S2A–E**). However, the EGRscore was not different between female and male (**Figure S2F**).

Stratification analyses were performed to evaluate the robustness of EGRscore. The HCC cohort was accordingly separated into EGRscore-high and EGRscore-low groups according to clinical stage, histological grade, the expression of AFP, vascular invasion, sex, and T stage. K-M curves demonstrated that EGRscore-high patients had a good prognosis than EGRscore-low patients regardless of clinical stage, histological grade, the expression of AFP, vascular invasion, sex, and T stage except for low expression of AFP (*p* = 0.053) and male (*p* = 0.061) (**Figures S1G–N**). K-M curves proved that high expression of EZH2, IGF2BP3, SUV39H2, TRMT6, YBX1, and YTHDF1 had worse OS than low expression (**Figure S3A**). Moreover, the results indicated that those six genes had a low level of mutations and copy number alterations (CNAs) (**Figures S3B, C**). To verify the EGR signature, we analyzed the expression of each gene by RT-qPCR in 13 pairs of human HCC and normal liver specimens. The result indicated that all those six genes were significantly elevated in HCC tissues (**Figure S3D**). In summary, our results suggested that EGRscore correlated with a worse clinical feature, OS, and could be an independent predictor for prognosis.

### External Validation Of The Prognostic Gene Signature

**Figure 2:**
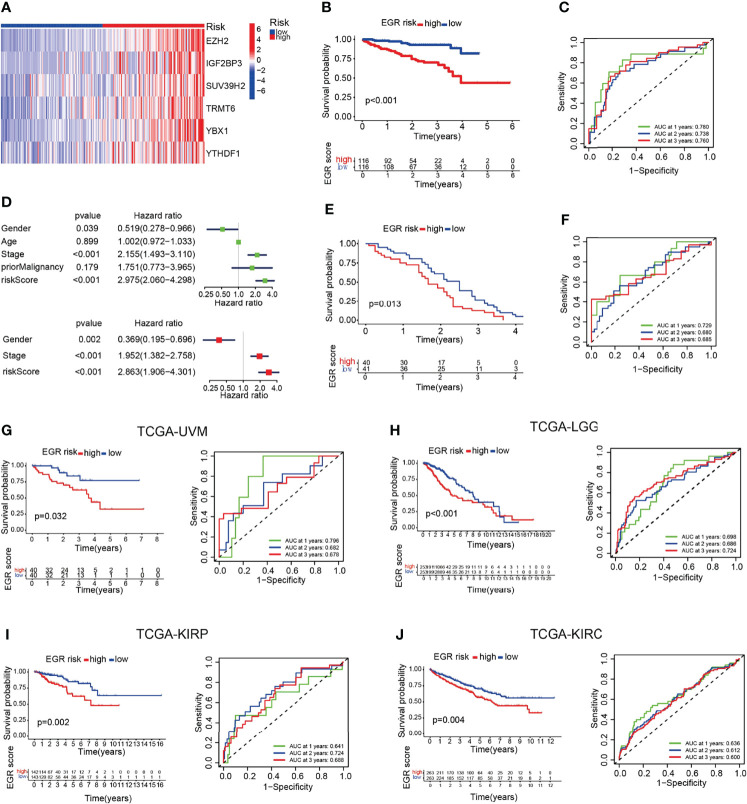
EGRscore is associated with overall survival across multiple independent cohorts. **(A)** Heatmap of genes consisted in EGR signature in normal and HCC patients. **(B)** Kaplan–Meier curves for the OS of patients between the high- and low-risk groups in the ICGC-JP cohort. **(C)** Time-dependent ROC curves demonstrated the predictive efficiency. **(D)** Univariate and multivariate Cox regression analyses for the EGRscore. **(E)** Kaplan–Meier curves for the OS of patients between the high- and low-risk groups in the GSE54236 cohort. **(F)** Time-dependent ROC curves demonstrated the predictive efficiency in the GSE54236 cohort. **(G)** Kaplan–Meier survival analysis and time-dependent ROC curves based on EGRscore in the TCGA-UVM cohort. **(H)** Kaplan–Meier survival analysis and time-dependent ROC curves based on EGRscore in the TCGA-LGG cohort. **(I)** Kaplan–Meier survival analysis and time-dependent ROC curves based on EGRscore in the TCGA-KIRP cohort. **(J)** Kaplan–Meier survival analysis and time-dependent ROC curves based on EGRscore in the TCGA-KIRC cohort.

To clarify the robustness of the EGR signature, we used the same formula to calculate the EGRscore of HCC patients in other HCC cohorts including the ICGC-LIRI-JP cohort and the GSE54236 cohort. Then, patients were divided into the EGRscore-high group and the EGRscore-low group with the median value as the cutoff value. The heatmap indicated that those six genes were upregulated in the EGRscore-high group compared with the EGRscore-low group (**Figure 2A**). The patients with low EGRscore in the ICGC-LIRI-JP cohort had a better survival rate than patients with a high EGRscore (*p* < 0.001), which was the same as the result in the TCGA-LIHC cohort (**Figure 2B**). The ROC curve suggested accurate prediction ability (1-year AUC = 0.791, 2-year AUC = 0.748, 3-year AUC = 0.77, **Figure 2C**). Furthermore, the univariate and multivariable Cox regression models were performed and the results indicated that EGRscore could be an independent predictor for prognosis (HR for EGRscore: 2.863, 95% CI: 1.906–4.301, *p* < 0.001, **Figure 2D**). The result was validated in the GEO database. We applied a K-M analysis and found that EGRscore-low patients had better OS than EGRscore-high patients in GSE54236 (**Figure 2E**) and the AUC value of the ROC curve for prognostic prediction was higher than 0.65 (**Figure 2F**).

To validate the fitness of EGR signature in other tumor cohorts, we used the same formula to calculate the EGRscore of other tumor cohorts in the TCGA database including UVM, LGG, KURP, and KIRC. The K-M analysis indicated that low-risk patients had better OS, and most of the AUC values were higher than 0.65 (**Figures 2H–J**). Collectively, these findings indicated that EGRscore could be applied to predict prognosis not only in the LIHC cohort but also in other tumor cohorts.

### Gene Mutation Analysis

**Figure 3:**
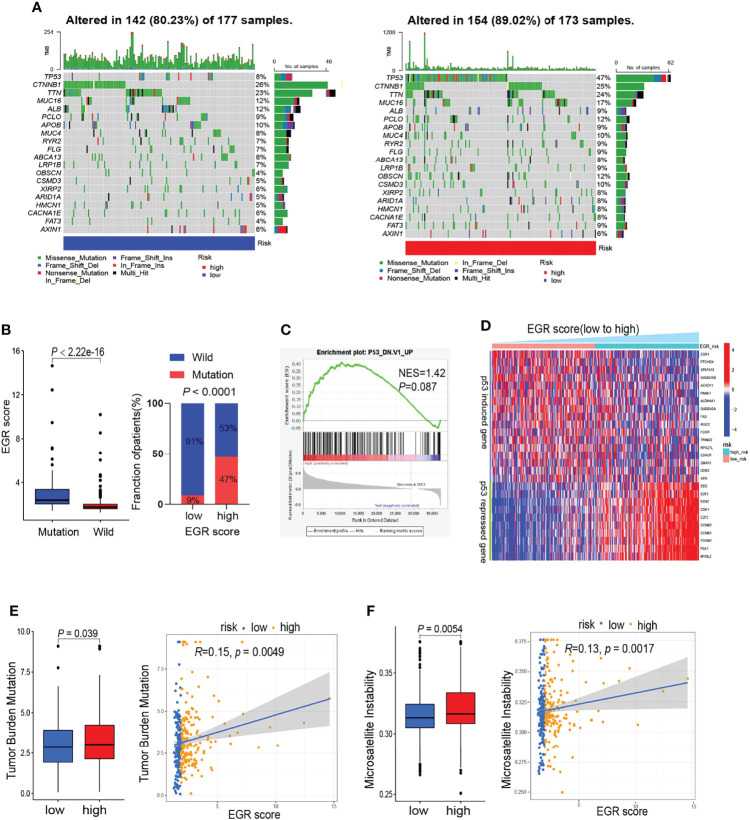
Comparison of mutation landscape between EGRscore-low and EGRscore-high tumors. **(A)** Waterfall maps between EGRscore-low and EGRscore-high HCC patients. **(B)** Proportion of the m6Ascore-high and m6Ascore-low group in patients that harbored wild-type or mutant TP53. **(C)** GSEA of genes upregulated in the NCI-60 panel of cell lines with mutated TP53. **(D)** Correlation of EGRscore and the expression of p53-induced genes or p53-suppressed genes in HCC patients. **(E)** Boxplot and correlation analysis of tumor burden mutation and EGRscore. **(F)** Boxplot and correlation analysis of microsatellite instability and EGRscore. ns, statistically insignificant.

To verify the differences in genomic mutations between high-risk and low-risk patients, we processed gene mutation data using the “maftools” R package. The waterfall maps indicated that TP53 is the most differentially mutant gene (**Figure 3A**). The boxplot suggested that patients with TP53 mutation had a higher EGRscore and high EGRscore associated with a high proportion of TP53 mutation (**Figure 3B**). GSEA suggested that EGRscore was positively associated with the activation of mutant TP53-induced genes (**Figure 3C**). p53-suppressed genes were upregulated in EGRscore-high HCC patients, while the p53-induced genes were downregulated (**Figure 3D**). The result indicated that the differences of mutation profiles, such as TP53, may contribute to the different OS between EGRscore-high and EGRscore-low HCC patients. Furthermore, the boxplot showed that EGRscore-high HCC patients had a higher TMB and MSI (**Figures 3E, F**) and EGRscore had a positive correlation with TMB and MSI.

### EGRscore Correlated With Immune Microenvironment

**Figure 4:**
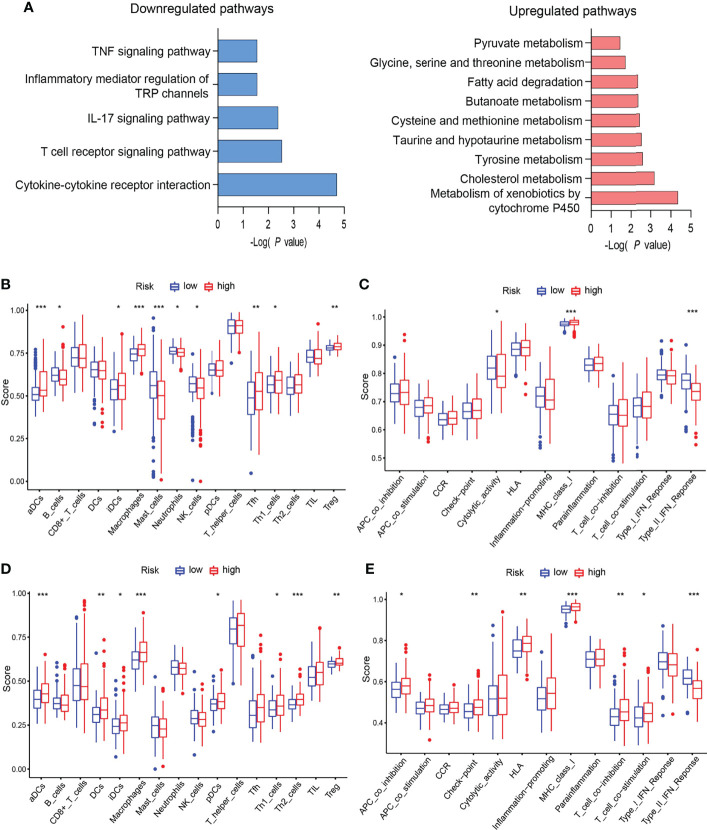
EGRscore correlated with immune microenvironment. **(A)** KEGG of DMGs in the high- and low-risk groups in the TCGA-LIHC cohort. **(B)** Comparison of immune cell infiltration in the high- and low-risk groups in the TCGA-LIHC cohort. **(C)** Comparison of immune function in the high- and low-risk groups in the TCGA-LIHC cohort. **(D)** Comparison of immune cell infiltration in the high- and low-risk groups in the ICGC-JIHC cohort. **(E)** Comparison of immune function in the high- and low-risk groups in the ICGC-JIHC cohort. Statistical significance was denoted with *(p < 0.05), **(p < 0.01), and ***(p < 0.001).

All of those six genes’ functions mainly depend on methylation. This urged us to explore the methylation effect of the EGR signature. *β* value was applied to indicate the extent of gene methylation, ranging from 0 to 1. Methylation data of HCC patients were downloaded from TCGA. Then, the “limma” package was applied to determine the differently methylated genes (DMGs). DMGs were applied to functional enrichment analysis. The KEGG results indicated that highly methylated genes driven by the signature were enriched in metabolism and lowly methylated genes were enriched in immune and inflammation pathways (**Figure 4A**).

Next, we explored the immune microenvironment in EGRscore-high patients and EGRscore-low patients. The ssGSEA method was applied for the RNA-seq data of the TCGA-LIHC cohort to evaluate immune cell infiltration and related function. The result demonstrated that the populations of immune cells promoting tumor killing effect, including B cells, NK cells, and neutrophils, were enriched in EGRscore-low patients and the populations of immune cells inhibiting tumor killing effect, including regulatory T cells (Tregs), were enriched in EGRscore-high patients (**Figure 4B**). Furthermore, immune function comparison indicated that EGRscore-low patients had higher cytolytic activity and type-II IFN response than EGRscore-high patients, whereas the opposite result was found for MHC class I (**Figure 4C**). Moreover, the ssGSEA method was also applied for the RNA-seq data of the ICGC-LIRI-JP cohort to evaluate immune cell infiltration and related function. The result was similar to the TCGA-LIHC cohort (**Figures 4D, E**). Taken together, the result suggested that EGRscore-low patients were correlated with immune microenvironment that promotes tumor killing.

### Potential Indicator for Immunotherapy

**Figure 5:**
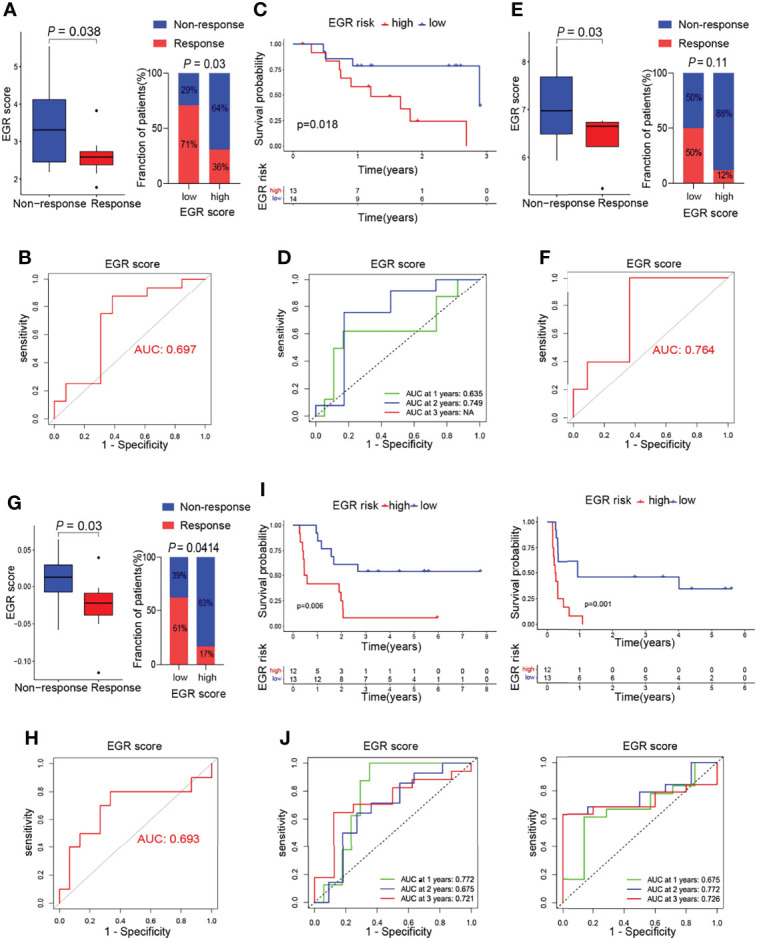
Association of m6Ascore and response to immunotherapy. **(A)** Boxplot of response of immunotherapy between EGRscore-low and EGRscore-high HCC patients. **(B)** ROC curves based on EGRscore in the GSE78220 cohort. **(C)** Kaplan–Meier survival analysis based on EGRscore in the GSE78220 cohort. **(D)** Time-dependent ROC curves based on EGRscore in the GSE78220 cohort. **(E)** Boxplot of response of immunotherapy between EGRscore-low and EGRscore-high HCC patients in the GSE126044 cohort. **(F)** ROC curves based on EGRscore in the GSE126044 cohort. **(G)** Boxplot of response of immunotherapy between EGRscore-low and EGRscore-high HCC patients in the GSE100797 cohort. **(H)** ROC curves based on EGRscore in the GSE100797 cohort. **(I)** Kaplan–Meier survival analysis for OS and DFS based on EGRscore in the GSE100797 cohort. **(J)** Time-dependent ROC curves for OS and DFS based on EGRscore in the GSE100797 cohort.

The above results indicated that low EGRscore correlated with immune microenvironment, and this prompted us to consider whether EGRscore could act as a biomarker to predict the response rate of immunotherapy. To test our hypothesis, tumor patients from the GSE78220, GSE126044, and GSE100797 cohorts who received immunotherapy were divided into EGRscore-high and -low patients with the median value as the cutoff value. The boxplots suggested that the response group had a low EGRscore compared to the non-response group in the GSE78220 cohort (**Figure 5A**), GSE126044 cohort (**Figure 5E**), and GSE100797 cohort (**Figure 5G**). Furthermore, the low EGRscore groups had a larger proportion of response rate (CR/PR) than high EGRscore groups in the GSE78220 cohort (**Figure 5A**), GSE126044 cohort (*p* = 0.11, **Figure 5E**), and GSE100797 cohort (*p* = 0.0414, **Figure 5G**). ROC curves of EGRscore in the GSE78220 cohort (**Figure 5B**), GSE126044 cohort (**Figure 5F**), and GSE100797 cohort (**Figure 5H**) indicated that the EGRscore had high accuracy in predicting the response of immunotherapy. K-M curves proved that EGRscore-high patients had a better OS than EGRscore-low patients in the GSE78220 cohort (**Figure 5C**) and GSE100797 cohort (**Figure 5I**), and a better disease-free survival (DFS) in the GSE100797 cohort (**Figure 5I**). TimeROC curves of EGRscore in the GSE78220 cohort (**Figure 5D**) and GSE100797 cohort (**Figure 5J**) indicated that the EGRscore had high accuracy and stability in predicting the OS of immunotherapy and DFS in the GSE100797 cohort (**Figure 5J**). Collectively, the result demonstrated that EGRscore could be a biomarker in predicting the response to immunotherapy.

### Performance of the EGRscore in Predicting the Response to Targeted Therapy and Chemotherapy

**Figure 6:**
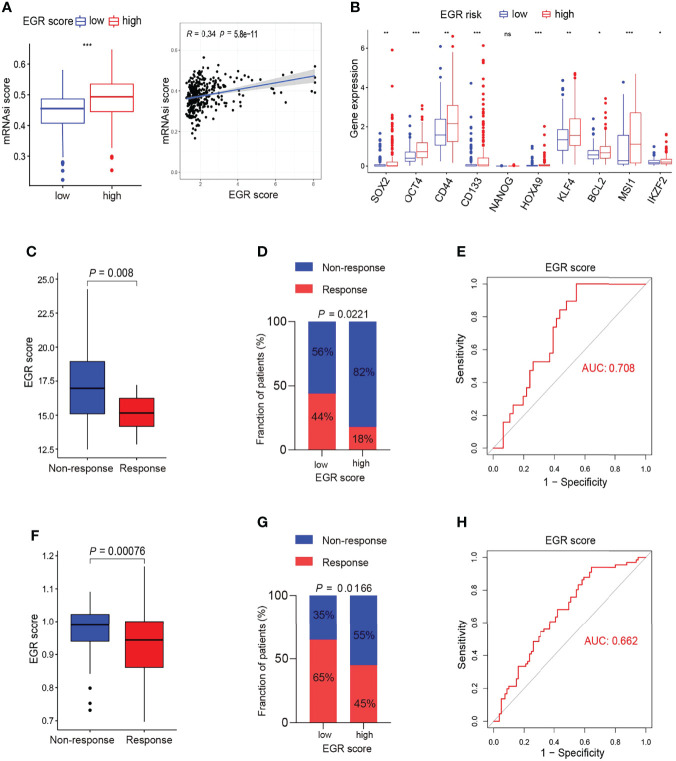
Association of m6Ascore and response to chemotherapy. **(A)** Boxplot of stemness score between EGRscore-low and EGRscore-high HCC patients. **(B)** Boxplot of genes associated with stemness between EGRscore-low and EGRscore-high HCC patients. **(C)** Boxplot of response of chemotherapy between EGRscore-low and EGRscore-high HCC patients. **(D)** Proportion of EGRscore-high and EGRscore-low group in patients that harbored a response or not in the GSE109211 cohort. **(E)** ROC curves based on EGRscore in the GSE109211 cohort. **(F)** Boxplot of response of TACE between EGRscore-low and EGRscore-high HCC patients. **(G)** Proportion of EGRscore-high and EGRscore-low group in patients that harbored a response or not in the GSE104580 cohort. **(H)** ROC curves based on EGRscore in the GSE104580 cohort. Statistical significance was denoted with *(p < 0.05), **(p < 0.01), and ***(p < 0.001).

The GSEA results demonstrated that the gene associated with high EGRscore significantly enriched in the DNA repair pathway, the PI3K/AKT pathway, and the MYC target pathway, which is associated with drug resistance (**Figures S4A–C**). Moreover, the boxplot indicated that genes that belong to those signaling pathways were upregulated in EGRscore-high patients (**Figures S4A–C**). ABC transporters and the EMT pathway had a close relationship with drug resistance, and we found that the genes associated with the pathway were upregulated in high-risk patients (**Figures S4D, E**). It is reported that stemness was associated with drug resistance. MRNAsi is an index to describe the stemness of tumor cells and ranges from 0 to 1 (16). The result suggested that EGRscore-high patients had a higher mRNAsi score than EGRscore-low patients (**Figure 6A**). The genes associated with stemness such as SOX2 and OCT4 had a higher expression in high-risk patients (**Figure 6B**). Thus, this urged us to prove whether EGRscore could be a biomarker for drug therapy resistance. Then, we applied the formula to calculate EGRscore in the GSE109211 cohort, in which patients were treated with resection/local ablation combined with targeted therapy. Of these patients, 67 received sorafenib therapy and 73 received placebo therapy. The boxplot indicated that responders had a lower EGRscore than non-responders (**Figure 6C**). The patients were divided into high and low risk according to the median value of EGRscore. The result demonstrated that EGRscore-low patients had a significantly high proportion of response rate than EGRscore-high patients treated with sorafenib (**Figure 6D**). We drew the ROC curve and the AUC value showed high prediction ability of the EGRscore to distinguish the response group from the non-response group (**Figure 6E**). Moreover, the “pRRophetic” package was applied to calculate the drug sensitivity (IC_50_) for TCGA-LIHC patients. The result indicated that high-risk patients had a higher IC_50_ and a slightly positive association between the IC_50_ of sorafenib and EGRscore (**Figures S4F, G**).

In order to expand the applicability of the signature, we applied the formula to calculate EGRscore in the GSE104580 cohort, in which patients were treated with transcatheter arterial chemoembolization (TACE). There were 147 patients, namely, 81 responders and 66 non-responders. The boxplot demonstrated that non-responders had a higher EGRscore than responders (**Figure 6F**). Taking the intermediate value as the cutoff value of EGRscore-high and EGRscore-low patients, the result demonstrated that EGRscore-low patients had a higher proportion of response rate than EGRscore-high patients (**Figure 6G**). The AUC value of the ROC curve was 0.662, which suggested the high predictive ability of the EGRscore to distinguish the response group from the non-response group treated with TACE (**Figure 6H**). To sum up, our research indicated that patients with a low EGRscore may benefit from patients who received targeted therapy and chemotherapy.

## Discussion

Epigenetic modification, which mainly included DNA methylation, m6A, m1C, and m5C, plays an important role in tumorigenesis and tumor progression. Previous research indicated that epigenetic modification could regulate the mutation and infiltration of immune cells and the activity of immune cells, thereby contributing to response to immunotherapy. However, how epigenetic modification affected immune function and tumor survival remains unclear. In this study, EGRs were used to construct a prognostic HCC signature that was associated with OS and tumor progression, and was able to distinguish the response of immunotherapy targeted therapy and chemotherapy.

The incidence of HCC ranks sixth and that of the cancer-related death ranks third in the world (1, 2). Up to now, the main treatment of solitary liver cancer is hepatectomy, but 70% of patients with primary liver cancer relapsed or metastasized within 5 years after treatment (17). Therefore, it is an important thing to distinguish OS of HCC patients and choose the corresponding treatment. Our study indicated that EGRscore-low HCC patients had a better OS in the TCGA cohort. The result was validated in the ICGC-LIRI-JP and GSE54236 cohorts. Furthermore, the result was validated in other tumor cohorts such as TCGA-LGG, TCGA-UVM, TCGA-KIRP, and TCGA-KIRC. Current studies found that m6A signature (18, 19) and m6A-associated lncRNA signature (20), DNA methylation signature (21), and m5C signature (22) could be used to distinguish the OS of HCC patients. These signatures have respectively analyzed the effects on the prognosis of HCC patients from different perspectives of epigenomics. However, this study depicted the association of all EGRs with the OS of HCC patients, so as to more accurately depict the relationship between the OS of HCC patients with epigenome.

The present studies have demonstrated a close relationship between methylation and immune microenvironment. Zhang et al. demonstrated that m6A regulator-mediated methylation modifies tumor microenvironment (TME) infiltration characterization in gastric cancer (23). Meng et al. showed that the DNA methylation regulator had a close relationship with TME and immune cell infiltration (24). In our research, KEGG indicated that the EGRscore-low group based on the prognostic signatures of six EGRs was enriched in immune-related pathways, such as IL-17 signaling pathways. Moreover, we found that the EGRscore-high group had a high infiltration level of macrophages and Tregs while the EGRscore-low group had a high infiltration level of B cells. An increasing number of studies demonstrated that B cells play a vital role in active immune microenvironment (25). Furthermore, our research found that TP53 mutation was different in EGRscore-high and -low patients and was more frequent in EGRscore-high patients. Previous studies indicated that mutation was associated with tumor immune evasion and resistance to immunotherapy. Sallman et al. indicated that TP53 mutation confers an immunosuppressive microenvironment in myelodysplastic syndromes and secondary acute myeloid leukemia (26). Dong et al. demonstrated that TP53 and KRAS mutation could serve as a potential biomarker for response to PD-1 immunotherapy (27). Mutation-induced tumor heterogeneity and clonal evolution result in the dynamic immune landscape of tumor tissues, which may mediate the effectiveness of immunotherapy (28). Furthermore, studies suggested that immune surveillance in the early stages of cancer may lead to the selection of evolved subclones with silent neoantigens due to promoter hypermethylation (29).

To clarify the role of EGR signature in predicting the response to immunotherapy, we applied three cohorts of patients who applied immune checkpoint inhibitors (ICIs) (30, 31) or adoptive cell therapy (ACT) treatment (32). EGRscore-low patients had a higher rate of response and the patients with response had a lower EGRscore. Moreover, K-M analysis suggested that EGRscore-low patients had a better OS in the GSE78220 and GSE100797 cohorts and a better DFS in the GSE100797 cohort, indicating that patients with a low EGRscore significantly benefited from immunotherapy and, thus, had a longer prognosis.

To elucidate the effect of EGR signature in predicting response to targeted therapy, we applied it to the BIOSTORM cohort (33) (GSE109211), in which patients were treated with resection/local ablation combined with sorafenib. We found that EGRscore-low patients had a higher rate of response and the patients with response had a lower EGRscore, indicating that patients who had a high EGRscore attained targeted therapy resistance.

To clarify the potential mechanism of our EGR signature with predictive value for immunotherapy and targeted therapy, we paid attention to differences in transcriptome data among groups. We found that the gene associated with stemness such as SOX2 and OCT4 in EGRscore-high patients was upregulated compared with EGRscore-low patients. A previous study found that Oncofetal HLF, reactivated by OCT4/SOX2, leads to stemness features contributing to primary sorafenib resistance (34). Stem cell status has been identified in different groups of EpCAM+ circulating tumor cells (CTCs), and the detection of this status has proved to be beneficial to evaluate the response to sorafenib (35).

DNA damage repair (DDR) has a dual role in tumor progression. DDR has the ability to inhibit the occurrence of tumors by maintaining the integrity of the genome. At the same time, in the process of treating induced tumors, cancer cells with sufficient DDR can outgrow tumor clones with defective DDR, therefore leading to chemotherapy resistance. The initial core of the DNA double-strand break (DSB) repair mechanism is the MRN complex composed of MRE11, RAD50, and NBN, while PALB2 and RAD51 cooperate with several other key regulators to contribute to the later stages of DDR (36). This research finds the upregulation of genes to be associated with DDR in EGRscore-high patients, demonstrating a possible link between EGRs and DDR in HCC. Furthermore, studies indicated that Myc signaling pathways, ABC transporters (37), and EMT (38) take part in chemotherapy resistance. Our study suggested that the expression of genes associated with Myc signaling pathways, ABC transporters, and EMT in the EGRscore-high group was higher compared with the EGRscore-low group.

EZH2, the most important gene in the EGR signature, plays a dual role, often synergistically, in both cancer cells and the TME. Studies have shown that tumor cell-mediated EZH2 exerts its epigenetic catalytic subunit methyltransferase ability, resulting in epigenetic changes in the TME into an immunosuppressive network. Overall, EZH2 has proven to be a driving force for immunoediting and resistance to tumor immunotherapy, primarily due to its epigenetic reprogramming of T-cell antigen-presenting genes. GSVA has demonstrated a negative association of EZH2 with major histocompatibility complex (MHC) class I antigen presentation molecules in HCC (39). A study indicates that EZH2 inhibits chemokines, such as CXCL9 and CXCL10, thus impairing the trafficking for CD8+ T cells in human ovarian carcinoma (40–42). Moreover, EZH2 secreted by immune cells could mediate chemokine, IFN-γ, Notch signaling pathways, etc. so as to affect T-cell trafficking (40), Th1/Th2 differentiation (43), and Teff cell survival (43). A number of clinical trials have been performed to explore the role of EZH2 associated with ICIs in tumor immunotherapy, such as NCT02601950 and NCT04703192.

This study also has limitations. How EGR signature affected immune microenvironment and immune evasion needs to be clarified. Further studies are needed to verify the effectiveness of EGRscore in predicting immunotherapeutic responses in clinical practice and to demonstrate the interaction of EGRs in mediating immune evasion.

In summary, EGRscore could be used to distinguish OS, tumor progression, mutation pattern, and immune microenvironment. Accurate diagnosis is useful for determining accurate treatment, and thus for improving HCC patient prognosis. Therefore, the present study contributes to improving HCC patient prognosis and predicting response to immunotherapy.

## Data Availability Statement

The datasets presented in this study can be found in online repositories. The names of the repository/repositories and accession number(s) can be found in the article/**Supplementary Material**.

## Author Contributions

JC, SW, FZ, and ZD designed the study. JC collected tissue samples and data. SW and FZ did the statistical analyses. JC, SW, and FZ prepared figures, reviewed the results, interpreted data, and wrote the manuscript. All authors have made an intellectual contribution to the manuscript and approved the submission.

## Funding

This work was supported by the National Natural Science Foundation of China (grant nos. 82072670 and 81871916), the Leading Project of the Science and Technology Committee of Shanghai Municipality (grant no. 21Y21900100), and the Project of Shanghai Municipal Health Commission (No. 202140269).

## Conflict of Interest

The authors declare that the research was conducted in the absence of any commercial or financial relationships that could be construed as a potential conflict of interest.

## Publisher’s Note

All claims expressed in this article are solely those of the authors and do not necessarily represent those of their affiliated organizations, or those of the publisher, the editors and the reviewers. Any product that may be evaluated in this article, or claim that may be made by its manufacturer, is not guaranteed or endorsed by the publisher.

## Supplementary Material

Supplementary Figure 1 Construction of nomogram. **(A)** Area Under the ROC Curve of 18-month survival; **(B)** The nomogram integrated the 6-gene signature, age, grade and stage. Each component gives points and the sum of the points calculated a linear predictor and overall survival.

Supplementary Figure 2 Correlation of EGR score with clinical characters. Boxplot of EGR score between stage1&2 and stage3&4 HCC patients **(A)**,grade1&2 and grade 3&4 HCC patients **(B)**, T1&2 and T3&4 HCC patients**(C)**,micro/macro vascular invasion and no vascular invasion HCC patients**(D)**,AFP<400 and AFP > = 400 HCC patients **(E)**, female and male HCC patients **( F)**; Kaplan-Meier survival analysis for based on EGR score in stage1&2 and stage3&4 HCC patients**(G)**, in grade1&2 and grade 3&4 HCC patients **(H)**, in grade1&2 and grade 3&4 HCC patients(H), in T1&2 and T3&4 HCC patients **(I)**, in micro/macro vascular invasion and no vascular invasion HCC patients **(J)**, in AFP<400 and AFP > = 400 HCC patients**(K)** and in female and male HCC patients **(L)**.

Supplementary Figure 3 Construction of EGR signature. **(A)** Kaplan-Meier survival analysis between EGR score high patients and EGR score low patients for EZH2, IGF2BP3, SUV39H2, TRMT6, YBX1 and YTHDF1; **(B)** Mutation rates of six genes (EZH2, IGF2BP3, SUV39H2, TRMT6, YBX1 and YTHDF1) in LIHC patients from the cBioPortal database; **(C)** CNVs of EZH2, IGF2BP3, SUV39H2, TRMT6, YBX1 and YTHDF1 in LIHC patients. D. mRNA expressions of MCT4 in 13 paired HCC and nontumor tissues were detected by RT-qPCR.

Supplementary Figure 4 Difference of Transcriptome difference between high EGR-score patients and low EGR-score patients. GSEA and boxplot of genes associated with DNA repair **(A)**, PI3K AKT MTOR signaling **(B)**, Myc targets **(C)** between high EGR-score patients and low EGR-score patients; Boxplot of genes associated with ABC transporters **(D)**, EMT **(E)** between high EGR-score patients and low EGR-score patients; Boxplot of IC50 of sorafenib between EGRscore-low and EGRscore-high HCC patients **(F)**; Correlation of IC50 of sorafenib with EGR score **(G)**.

## Abbreviations

EGR, epigenetic regulator; LIHC, liver hepatocellular carcinoma; HCC, hepatocellular carcinoma; OS, overall survival; CNAs, copy number alterations; UVM, uveal melanoma; TMB, tumor mutational burden; MSI, microsatellite instability; PDL-1, programmed cell death 1 ligand 1; TCGA, The Cancer Genome Atlas; GEO, Gene Expression Omnibus; ICGC, International Cancer Genome Consortium; LGG, brain lower grade glioma; KIRP, kidney renal papillary cell carcinoma; KIRC, kidney renal clear cell carcinoma; DEGs, differentially expressed genes; FDR, false discovery rate; LASSO, least absolute shrinkage and selection operator; EZH2, enhancer of zeste 2 polycomb repressive complex 2 subunit; TRMT6, tRNA methyltransferase 6 non-catalytic subunit; YBX1, Y-box binding protein 1; IGF2BP3, insulin-like growth factor 2 mRNA binding protein 3; SUV39H2, SUV39H2 histone lysine methyltransferase; YTHDF1, YTH N6-methyladenosine RNA binding protein 1; KEGG, Kyoto Encyclopedia of Genes and Genomes; GSEA, Gene Set Enrichment Analysis; ssGSEA, single-sample gene set enrichment analysis; ROC, receiver operating characteristic; AUC, area under the curve; CTLA-4, cytotoxic T-lymphocyte associated protein 4; AFP, alpha fetoprotein; DMGs, differently methylated genes; Tregs, regulatory T cells; DFS, disease-free survival; EMT, epithelial–mesenchymal transition; ABC transporter, ATP-binding cassette transporter; SOX2, SRY-box transcription factor 2; OCT4, organic cation/carnitine transporter4; TACE, transcatheter arterial chemoembolization; ICIs, immune checkpoint inhibitors; ACT, adoptive cell therapy; MHC, major histocompatibility complex; TME, tumor microenvironment; GSVA, gene set variance analysis; CXCL9, C-X-C motif chemokine ligand 9; CXCL10, C-X-C motif chemokine ligand 10; CTCs, circulating tumor cells; DDR, DNA damage repair; MRE11, MRE11 homolog, double strand break repair nuclease; RAD50, RAD50 double strand break repair protein; NBN, nibrin.

## Glossary

**Table:**

EGR	epigenetic regulator
LIHC	liver hepatocellular carcinoma
HCC	hepatocellular carcinoma
OS	overall survival
CNAs	copy number alterations
UVM	uveal melanoma
TMB	tumor mutational burden
MSI	microsatellite instability
PDL-1	programmed cell death 1 ligand 1
TCGA	The Cancer Genome Atlas
GEO	Gene Expression Omnibus
ICGC	International Cancer Genome Consortium
LGG	brain lower grade glioma
KIRP	kidney renal papillary cell carcinoma
KIRC	kidney renal clear cell carcinoma
DEGs	differentially expressed genes
FDR	false discovery rate
LASSO	least absolute shrinkage and selection operator
EZH2	enhancer of zeste 2 polycomb repressive complex 2 subunit
TRMT6	tRNA methyltransferase 6 non-catalytic subunit
YBX1	Y-box binding protein 1
IGF2BP3	insulin-like growth factor 2 mRNA binding protein 3
SUV39H2	SUV39H2 histone lysine methyltransferase
YTHDF1	YTH N6-methyladenosine RNA binding protein 1
KEGG	Kyoto Encyclopedia of Genes and Genomes
GSEA	Gene Set Enrichment Analysis
ssGSEA	single-sample gene set enrichment analysis
ROC	receiver operating characteristic
AUC	area under the curve
CTLA-4	cytotoxic T-lymphocyte associated protein 4
AFP	alpha fetoprotein
DMGs	differently methylated genes
Tregs	regulatory T cells
DFS	disease-free survival
EMT	epithelial–mesenchymal transition
ABC transporter	ATP-binding cassette transporter
SOX2	SRY-box transcription factor 2
OCT4	organic cation/carnitine transporter4
TACE	transcatheter arterial chemoembolization
ICIs	immune checkpoint inhibitors
ACT	adoptive cell therapy
MHC	major histocompatibility complex
TME	tumor microenvironment
GSVA	gene set variance analysis
CXCL9	C-X-C motif chemokine ligand 9
CXCL10	C-X-C motif chemokine ligand 10
CTCs	circulating tumor cells
DDR	DNA damage repair
MRE11	MRE11 homolog, double strand break repair nuclease
RAD50	RAD50 double strand break repair protein
NBN	nibrin
