# Supplementary figures and images for: Construction and Validation of an Epigenetic Regulator Signature as A Novel Biomarker For Prognosis, Immunotherapy, And Chemotherapy In Hepatocellular Carcinoma
Source: Front Immunol. 2022 Jul 14;13:952413. doi: 10.3389/fimmu.2022.952413 (PMC9330038; doi:10.3389/fimmu.2022.952413)

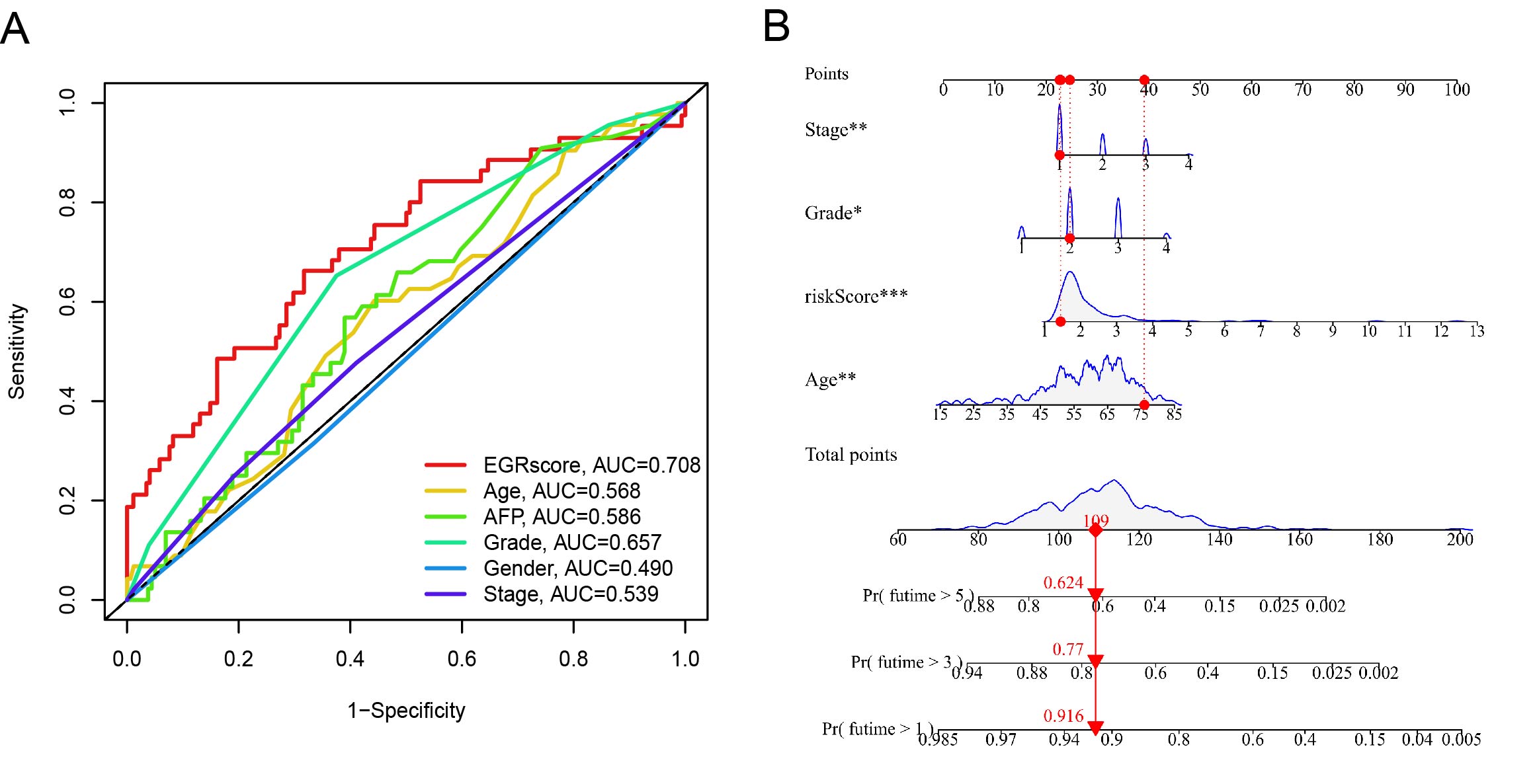

Supplement: Supplementary Figure 1 — Construction of nomogram. (A) Area Under the ROC Curve of 18-month survival; (B) The nomogram integrated the 6-gene signature, age, grade and stage. Each component gives points and the sum of the points calculated a linear predictor and overall survival. [file Image_1.jpeg]

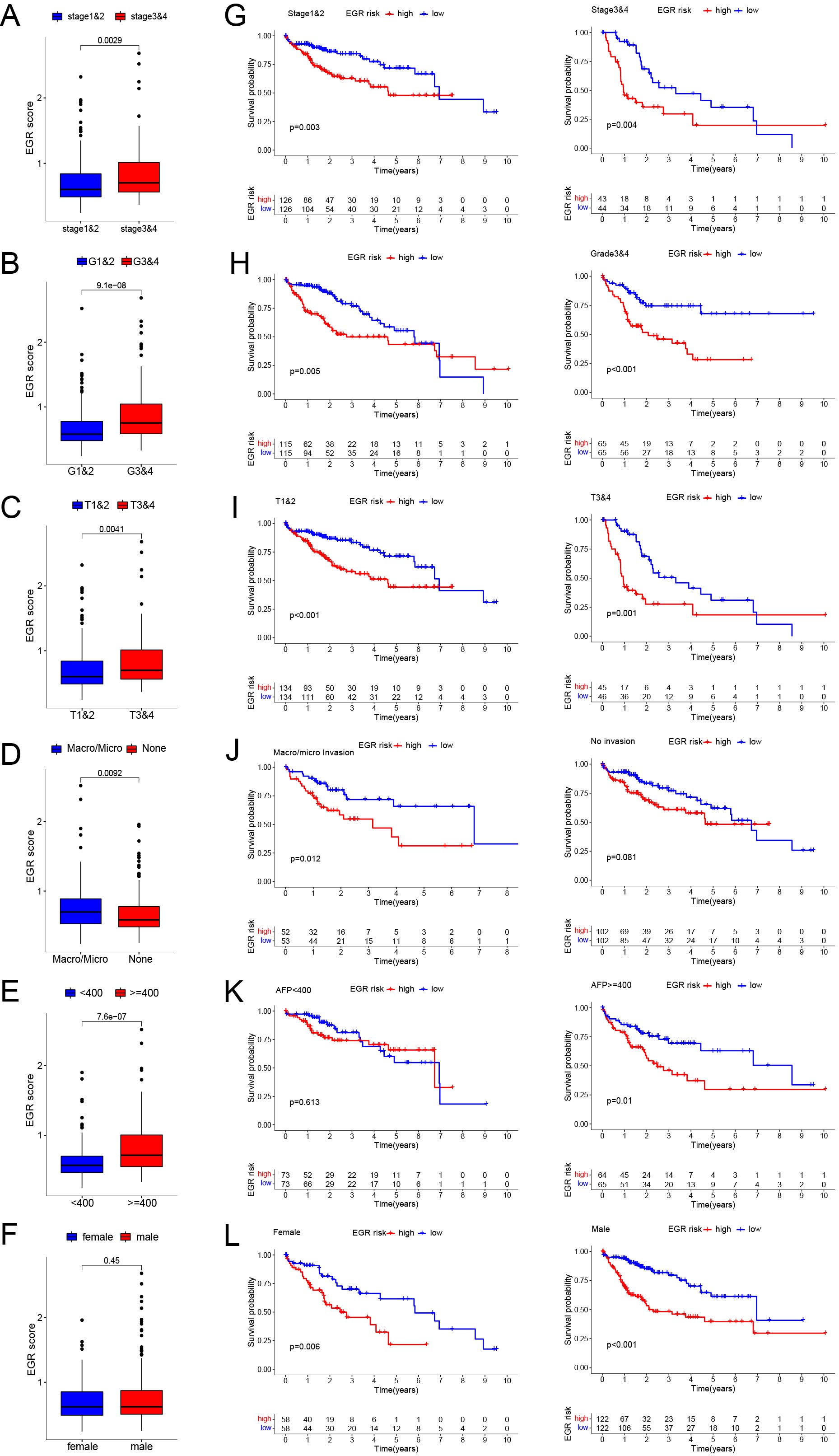

Supplement: Supplementary Figure 2 — Correlation of EGR score with clinical characters. Boxplot of EGR score between stage1&2 and stage3&4 HCC patients (A),grade1&2 and grade 3&4 HCC patients (B), T1&2 and T3&4 HCC patients(C),micro/macro vascular invasion and no vascular invasion HCC patients(D),AFP<400 and AFP > = 400 HCC patients (E), female and male HCC patients ( F); Kaplan-Meier survival analysis for based on EGR score in stage1&2 and stage3&4 HCC patients(G), in grade1&2 and grade 3&4 HCC patients (H), in grade1&2 and grade 3&4 HCC patients(H), in T1&2 and T3&4 HCC patients (I), in micro/macro vascular invasion and no vascular invasion HCC patients (J), in AFP<400 and AFP > = 400 HCC patients(K) and in female and male HCC patients (L). [file Image_2.jpeg]

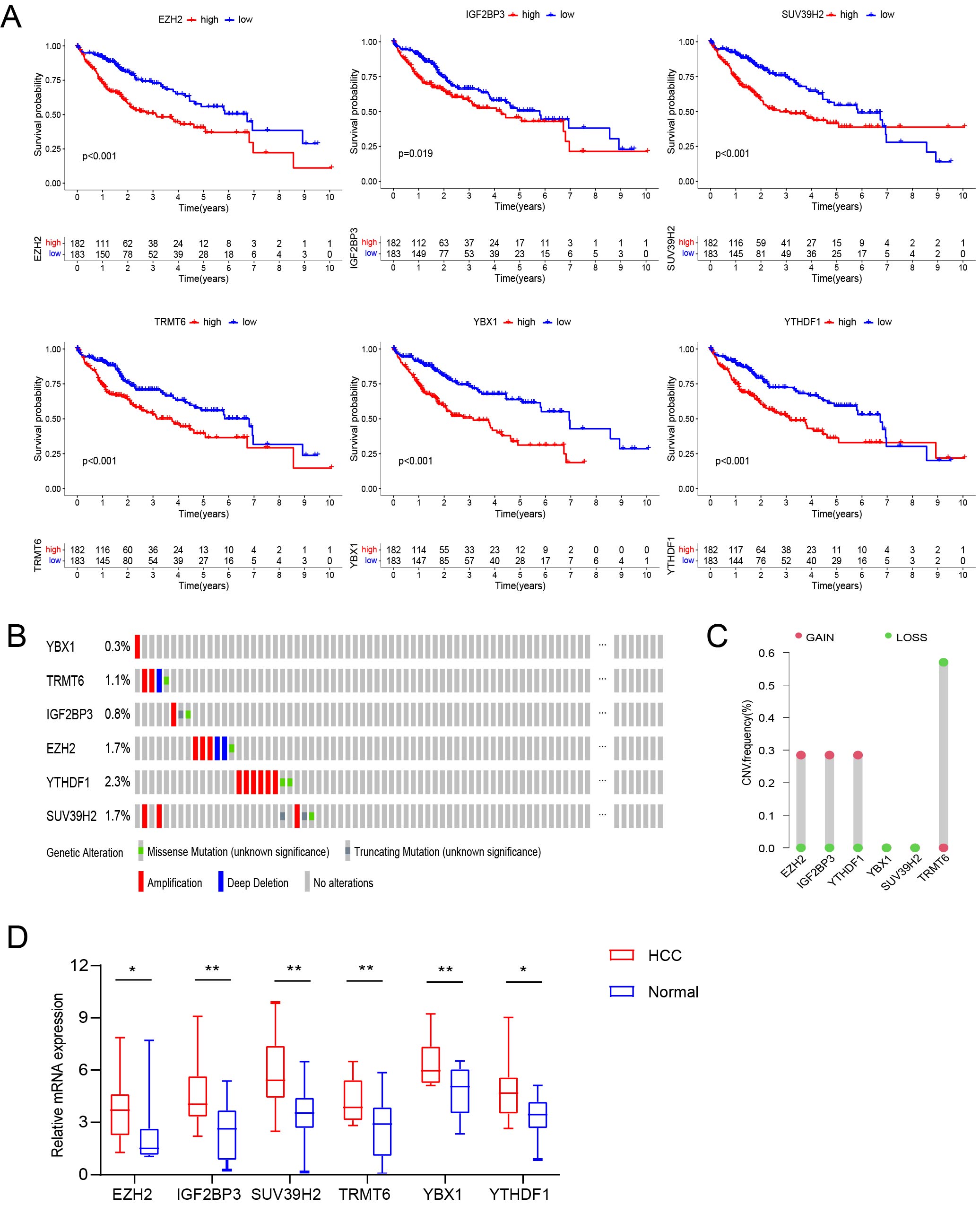

Supplement: Supplementary Figure 3 — Construction of EGR signature. (A) Kaplan-Meier survival analysis between EGR score high patients and EGR score low patients for EZH2, IGF2BP3, SUV39H2, TRMT6, YBX1 and YTHDF1; (B) Mutation rates of six genes (EZH2, IGF2BP3, SUV39H2, TRMT6, YBX1 and YTHDF1) in LIHC patients from the cBioPortal database; (C) CNVs of EZH2, IGF2BP3, SUV39H2, TRMT6, YBX1 and YTHDF1 in LIHC patients. D. mRNA expressions of MCT4 in 13 paired HCC and nontumor tissues were detected by RT-qPCR. [file Image_3.jpeg]

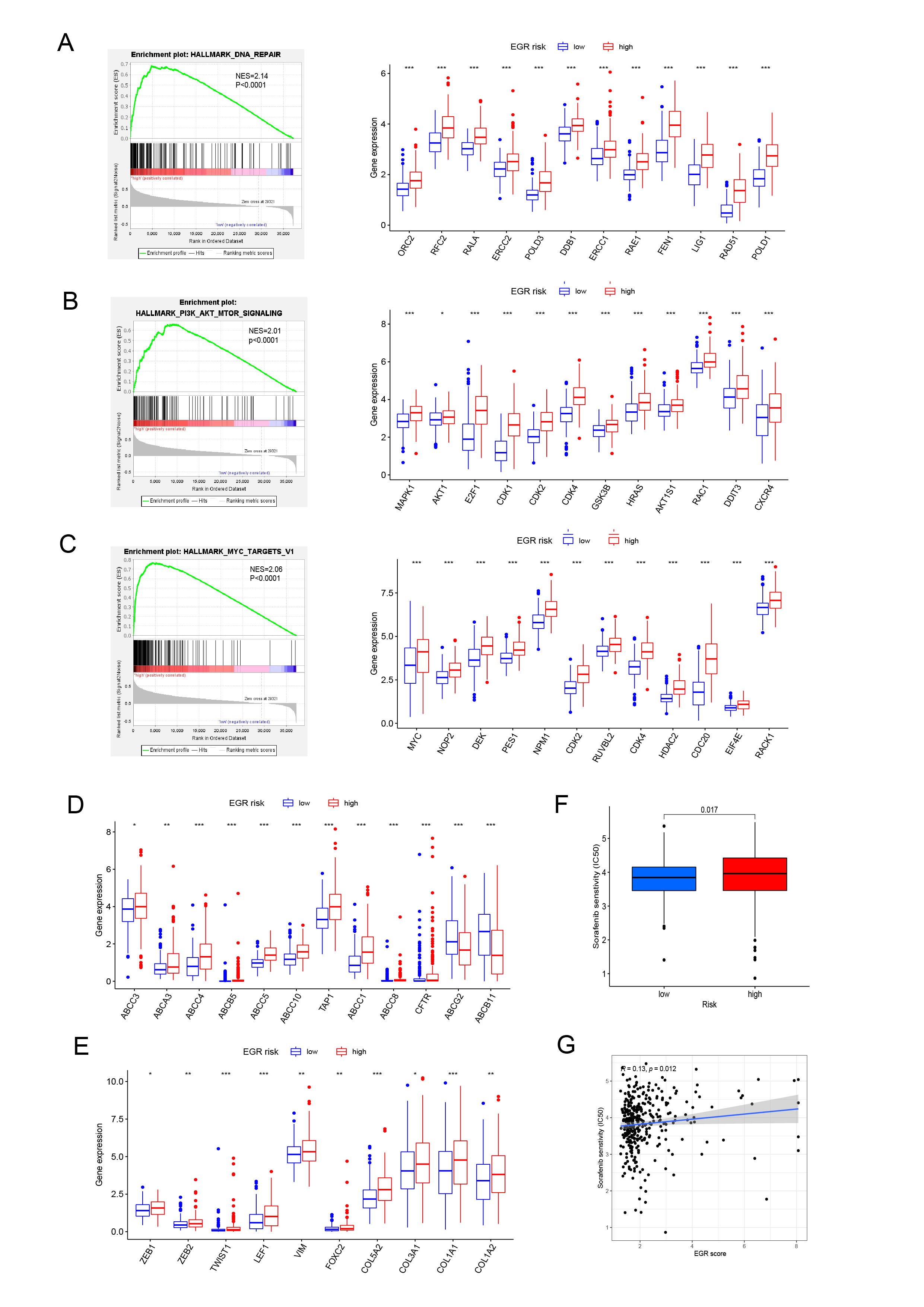

Supplement: Supplementary Figure 4 — Difference of Transcriptome difference between high EGR-score patients and low EGR-score patients. GSEA and boxplot of genes associated with DNA repair (A), PI3K AKT MTOR signaling (B), Myc targets (C) between high EGR-score patients and low EGR-score patients; Boxplot of genes associated with ABC transporters (D), EMT (E) between high EGR-score patients and low EGR-score patients; Boxplot of IC50 of sorafenib between EGRscore-low and EGRscore-high HCC patients (F); Correlation of IC50 of sorafenib with EGR score (G). [file Image_4.jpeg]
