# Supplementary material for: Construction and Validation of an Epigenetic Regulator Signature as A Novel Biomarker For Prognosis, Immunotherapy, And Chemotherapy In Hepatocellular Carcinoma
Source: Front Immunol. 2022 Jul 14;13:952413. doi: 10.3389/fimmu.2022.952413 (PMC9330038; doi:10.3389/fimmu.2022.952413)
Supplement: Supplementary file 6 [file Table_2.docx]

| Basic information of associated cohorts induced in this study. | | | | | | | | |
| --- | --- | --- | --- | --- | --- | --- | --- | --- |
| Dataset | No. of cases | Gender | Age | T Stage | N Stage | M Stage | Stage | Platform |
| TCGA-LIHC | 374 | M: 253 F: 121 | <60:169,  >=60:204 | 1-2:278  3-4:93 | 0:254  1:4 | 0:268  1:4 | I/II:260  III/IV:90 | Illumina |
| TCGA-UVM | 80 | M: 45 F: 35 | <60:36,  >=60:44 | 1-2:7  3-4:74 | 0:76  1:0 | 0:73  1:3 | I/II:36  III/IV:44 | Illumina |
| TCGA-LGG | 511 | M: 282 F: 228 | <60:441,  >=60:69 | NA | NA | NA | NA | Illumina |
| TCGA-KIRP | 288 | M: 212 F: 76 | <60:119,  >=60:166 | 1-2:165  3-4:36 | 0:132  1-2:21 | 0:199  1:9 | I/II:159  III/IV:39 | Illumina |
| TCGA-KIRC | 526 | M: 342 F: 184 | <60:242,  >=60:284 | 1-2:337  3-4:189 | 0:239  1:16 | 0:416  1:78 | I/II:319  III/IV:204 | Illumina |
| ICGC-LIRI-JP | 243 | NA | NA | NA | NA | NA | I/II:146  III/IV:97 |  |
| GSE54236 | 81 | M: 64 F: 17 | NA | NA | NA | NA | NA | Agilent-014850 Whole Human Genome Microarray 4x44K G4112 |
| GSE78220 | 27 | M: 19 F: 8 | <60:12,  >=60:15 | NA | NA | NA | NA | Illumina HiSeq2000 |
| GSE126044 | 16 | NA | NA | NA | NA | NA | NA | Illumina HiSeq 2500 |
| GSE100797 | 25 | NA | NA | NA | NA | NA | NA | Illumina HiSeq 2000 |
| GSE109211 | 140 | NA | NA | NA | NA | NA | NA | Illumina HumanHT-12 WG-DASL V4.0 expression beadchip |
| GSE104580 | 147 | NA | NA | NA | NA | NA | NA | Affymetrix Human Genome U133 Plus 2.0 Array |
